# The claustrum in review

**DOI:** 10.3389/fnsys.2014.00048

**Published:** 2014-04-04

**Authors:** Brian N. Mathur

**Affiliations:** Department of Pharmacology, University of Maryland School of Medicine Baltimore, MD, USA

**Keywords:** claustrum, cerebral cortex, connections, function, attention

## Abstract

The claustrum is among the most enigmatic of all prominent mammalian brain structures. Since the 19th century, a wealth of data has amassed on this forebrain nucleus. However, much of this data is disparate and contentious; conflicting views regarding the claustrum’s structural definitions and possible functions abound. This review synthesizes historical and recent claustrum studies with the purpose of formulating an acceptable description of its structural properties. Integrating extant anatomical and functional literature with theorized functions of the claustrum, new visions of how this structure may be contributing to cognition and action are discussed.

## Introduction

Despite centuries of investigation, the claustrum continues to evade complete structural and functional characterization. In their comprehensive study of comparative neuronanatomy, Ariëns Kappers et al. (1936, 1960) described the “Problem of the Claustrum”, which largely centered on the debate over claustral gross and fine morphology, connectivity, and function. Though much has been learned since this time through the application of modern neuroscience techniques, several problems persist. Notably, the exact borders of the claustrum have been called into question, an issue that carries implications for connectivity and functional conclusions, including those based on functional imaging, lesioning, electrophysiological single-unit recording, and optogenetic approaches. Thus, it is essential that a cogent structural definition should be established to inform functional conclusions for the ultimate purpose of solving the “problem” of the claustrum.

Contributing to the mysterious nature of the claustrum is the varied nomenclature used since its initial description. This structure was originally named the “nucleus taeniaformis” by the French comparative anatomist Vicq d’Azyr around the turn of the 18th century, and would soon thereafter be renamed the “claustrum” by Burdach (Rae, 1954). “Claustrum” often collectively refers to both “dorsal claustrum”, otherwise known as the “insular claustrum” or “field 8” (Brodmann, 1909), and a structure that is ventrally contiguous called the “ventral claustrum”, otherwise known as the “endopiriform nucleus” (Loo, 1931), or “claustrum ventrale” (Druga, 1966; Druga et al., 1990, 1993). The endopiriform nucleus can further be subdivided into the dorsal endopiriform nucleus and the ventral endopiriform nucleus (Paxinos and Watson, 1997); the ventral endopiriform nucleus, which also is known as the “claustrum praefiriforme” (Brockhaus, 1940; Narkiewicz, 1964), is very poorly described. The term claustrum will be used to refer to the “dorsal claustrum” while the term endopiriform nucleus will be used to refer to the “ventral claustrum” hereafter, following current conventions.

## Morphology

### Macroscopic morphology

Structurally, the claustrum is a long, band-like gray matter structure in the ventrolateral telencephalon of all therian mammals (marsupials and placentals), and arguably in monotremes (Loo, 1931; Butler et al., 2002; Ashwell et al., 2004). Therian mammals can be divided into two groups based on claustrum morphologies: species lacking an extreme capsule of white matter (hedgehog, bat, mouse, and rat), and species possessing an extreme capsule of white matter (guinea pig, rabbit, cow, carnivores, non-human primates, and human).

Among species lacking an extreme capsule, the structural organization of the claustrum has been most heavily studied in the rat. Nonetheless, views on the structural boundaries of the claustrum in this species (and other extreme capsule-lacking species) have been historically inconsistent, in part because no claustrum-specific neuroanatomical marker had been identified. It is, therefore, not surprising that accounts of claustral borders for the rat vary between brain atlases (Swanson, 2004; Paxinos and Watson, 2007), as well as across various primary research sources (Krettek and Price, 1977; Bayer and Altman, 1991; Druga et al., 1993; Kowiański et al., 1999; McKenna and Vertes, 2004; Mathur et al., 2009). Paxinos and Watson do not cite a source for their definition, but Swanson cites Krettek and Price (1977, 1978). Krettek and Price (1977) define the claustrum as extending along the entire rostrocaudal length of the striatum, where it resides immediately adjacent to the medially-lying external capsule (EC). However, both the Paxinos and Swanson atlases, as well as primary literature sources such as McKenna and Vertes (2004), extend the claustrum much further rostrally than the descriptions of Krettek and Price (1977) or Bayer and Altman (1991), well into the frontal pole where it lies immediately ventrolateral to the forceps minor. The reason for this rostral extension is unclear. Paxinos and Watson (2007) noted in their atlas a new, dorsally-lying component to the claustrum, which they termed the “dorsal claustrum”. This area has yet to be examined.

In species possessing an extreme capsule, the structural organization of the claustrum is ostensibly easier to define; it is historically defined as the thin strip of gray matter interposed between the striatum and the insular cortex. Consistent with the definition of its name, meaning “hidden” or “enclosed space”, the claustrum appears completely enveloped by the medially-lying EC and the laterally-lying extreme capsule of white matter. The arbitrary border created by this surrounding white matter and the gray matter that is enclosed within thus defines the structural boundaries of the claustrum. In humans, as an example of a species possessing an extreme capsule, the claustrum is present along the entire rostrocaudal extent of the striatum (Jennes et al., 1995). Dorsoventrally, the claustrum extends along the entire medial face of the adjacent insula. Along this dorsoventral axis, the claustrum undulates slightly, following the contours of the insula. From an oblique angle, then, the claustrum appears as a wavy sheet of gray matter (Rae, 1954).

### New Macroscopic Morphological Definitions

**Figure 1:**
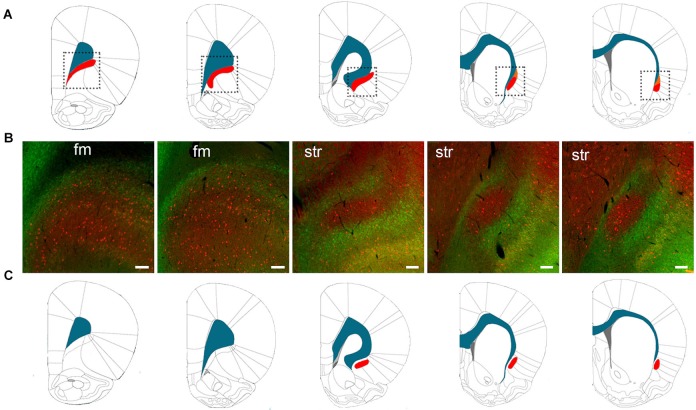
**(A)** The structural boundaries of the rat claustrum (shown in red) and its proximity to the white matter (shown in blue) of the forceps minor (fm) and EC as defined by Paxinos and Watson (2007) (used with permission from Elsevier). The dotted lines in **(A)** indicate the regions depicted in **(B)**, which shows immunohistochemical staining for parvalbumin (PV) (red) and crystallin mu (Crym), a marker of insular cortex (staining originally published in Mathur et al., 2009). At levels of the striatum (str), the body of the claustrum is labeled by PV-immunoreactivity (-ir) and surrounded by Crym-ir, indicating that the claustrum is not immediately juxtaposed to the white matter. At the level of the fm, however, PV-ir and Crym-ir does not reveal structural boundaries of the claustrum as defined by Paxinos and Watson (2007). **(C)** The structural boundaries of the claustrum redrawn to depict the definition based on PV-ir and Crym-ir, as well as G protein gamma 2 subunit (Gng2)-ir (Mathur et al., 2009). Scale bars: 200 μm for fm sections; 100 μm for str sections.

The varying definitions of the borders of the rat claustrum arose from the absence of a claustrum-specific marker protein. In 2009, however, we found a claustral marker, G protein gamma 2 subunit (Gng2), to be enriched in the claustrum and in register with parvalbumin-immunoreactive (PV-ir) neuropil (Mathur et al., 2009). The Gng2/parvalbumin (PV)-based definition of the claustrum alters the borders proposed by rat brain atlases. Paxinos and Watson (2007), for example, show the claustrum to extend well beyond the rostral pole of the striatum, lying ventrolaterally to the forceps minor (Figure 1A). However, Gng2 and PV immunostaining data, AChE and cytochrome oxidase histochemistry and tract tracing data indicate that the rostral most extension of the claustrum does not actually reach beyond the rostral pole of the striatum (Mathur et al., 2009; Smith and Alloway, 2010). Moreover, co-immunostaining for PV and crystallin mu (Crym), a marker of insular layer VI (Arlotta et al., 2005), does not readily reveal the presence of the claustrum at levels rostral to the striatum (Figure 1B; see Mathur et al., 2009 for methods). This suggests the rat claustrum is only present at striatal levels (Figure 1C). The PV and Crym immunohistochemistry data also indicate that the claustrum is situated not between the EC and the insular cortex, but embedded within layer VI of insular cortex. The PV-positive cloud of neuropil that defines the claustrum lies lateral to the Crym-ir deep layer VI cells of the insula, and not immediately adjacent to the white matter of the EC as is depicted by Paxinos and Watson (2007), for example (Figures 1A–C).

For the rat claustrum then, protein expression data finally exists to define this structure: this Gng2/PV-based definition excludes the pregenual extent of the claustrum proposed by atlases, which derive their definition from Krettek and Price (1977). It should be noted that the manuscript by Krettek and Price (1977) is focused on the prefrontal cortex, only tangentially mentions the claustrum, and does not provide a rationale for the anatomical definitions of this nucleus. As such, the borders outlined in Figure 1C represent the first empirical anatomical definition for the claustrum.

### Microscopic morphology

Following early investigations of the gross anatomy of the claustrum by Dejerine (1895) and others, Rae (1954) published a careful microscopic analysis in the human. Using silver impregnation studies, he found that the interface of capsular fibers and the gray matter body of the claustrum, which represents the structural boundaries of the claustrum, are not clearly demarcated; the dense collection of cell bodies and fibers in the core of the claustrum gradually change toward the border with capsular fibers. Specifically, he observed that fusiform-shaped cells, became more prevalent toward the perimeter of the claustrum. Interestingly, both Meynert (1884) and Brodmann (1909) also observed the prevalence of fusiform somata in the claustral perimeter, which they found to be enriched in the insular cortex. It has yet to be investigated whether the fusiform somata on the claustral periphery are actually insular cortex cells, as would be suggested by the finding that the claustrum is surrounded by insular cortex (Mathur et al., 2009). Besides the heterogeneous distribution of fusiform cells in the claustrum, Rae (1954) found a homogenous distribution of other cell types within the claustrum, including ovoid, triangular, and polygonal types.

The significance of these ovoid, triangular, and polygonal types remains unclear as golgi impregnation analysis of human tissue has only defined two types of neurons, type I and type II (Braak and Braak, 1982). Golgi type I neurons comprise roughly 85% of all claustral neurons and are evenly distributed throughout the body of the claustrum (Braak and Braak, 1982; Spahn and Braak, 1985; Sherk, 1986). They have spiny dendrites with axons projecting out of the claustrum, and have cell body diameters of 15–29 nm. The type I neurons represent the excitatory neurons that send projections to and receive projections from the cortex. A combined neuronal tract tracer and in situ hybridization study in rat demonstrated that the claustral projection neurons express the gene encoding the vesicular glutamate transporter (Vglut) 2 (Hur and Zaborszky, 2005). Because Vgluts (1 and 2) are considered to be unambiguous markers of cells that use glutamate as a neurotransmitter, it can reasonably be inferred that these claustral projection neurons are glutamatergic.

The less common Golgi type II neurons, comprising the remaining 15% of claustral cells, have cell body diameters of 10–15 nm, are aspiny, and have axons that do not project outside the body of the claustrum, as evidenced by human and cat studies (LeVay and Sherk, 1981; Braak and Braak, 1982). Type II neurons are therefore thought to be interneurons. This suggestion is bolstered by the fact that retrograde tract tracing studies have found that these cells do not accumulate tracer. These cells may express one of three types of calcium-binding proteins: PV, calbindin (CB), and calretinin (CR; Druga et al., 1993; Reynhout and Baizer, 1999). The rat claustrum is rich in PV-positive interneurons, but relatively poor in CB and CR-positive interneurons (Druga et al., 1993; Paxinos et al., 1999). Immunohistochemical analysis reveals a dense cloud of PV-ir neuropil with interspersed PV-ir somata in the rat claustrum, while a plexus of neuropil rich in CR-ir surrounds the claustrum in what appears as a ring around the nucleus. However, overlap exists between the PV-ir and the CR-ir plexuses.

Unlike the rat claustrum, the non-human primate claustrum has a much more homogenous distribution of interneuron populations compared to the rat, although the density between these populations varies. Reynhout and Baizer (1999) found PV-ir neurons to be large, multipolar cells with smooth dendrites in the macaque (*Macaca fascicularis*). In comparison, CR-ir cells are smaller, have elongated somata, are bipolar, and exhibit beaded dendrites. The CB-ir neurons were shown to exist in three forms: a dense population with small cell bodies and winding dendrites, a second multipolar type not unlike the PV-positive neurons, and a third bipolar type resembling the CR-positive neurons. Similar cell types have been observed in several different species, including human (Brand, 1981; LeVay and Sherk, 1981; Braak and Braak, 1982; Mamos, 1984; Mamos et al., 1986; Rahman and Baizer, 2007; Hinova-Palova et al., 2013).

Not unlike the cortex, then, the claustrum is composed of inhibitory-like interneurons and excitatory projection neurons; it is highly likely that several subclasses of both types of neurons await identification. Unlike the cortex, the claustrum does not exhibit a layered organization. Moreover, the dendrites of the type I projection neurons are not oriented in any specific direction, and these neurons express Vglut2, which is typically restricted to subcortical cells (Hur and Zaborszky, 2005). This suggests that the claustrum is a subcortical, or at least non-cortical, structure despite its physical apposition to and high connectivity with cortex, as well as its presence of inhibitory interneurons and excitatory projection neurons.

## Ontogeny

Early in the 20th century, the ontogenic and phylogenic derivations of the claustrum were intensely contested by several comparative anatomists. Investigators agreed that the claustrum is of pallial derivation. However, a dispute arose over whether the claustrum should be considered a derivative of cortex or a subcortical (albeit pallial) structure. Holl (1899) viewed the claustrum as a doubling of the insular cortex, and Smith (1910, 1917) later independently concluded that the claustrum derived from the upturned aspect of the piriform cortex. This notion of a doubling of adjacent cortex was also supported by Brodmann (1909) and others who concluded that the claustrum is cortical in origin. De Vries shared this view, but submitted that this did not necessarily mean that the claustrum was derived from cortex (Ariëns Kappers et al., 1936, 1960).

Carrying the cortical derivation hypothesis further, Sonntag and Woollard (1925) noted the resemblance of layer VI cells of the insular cortex and claustral cells in the aardvark. They concluded that the deepest layer of insular cortex is a “two-layered lamina multiformis” that is separated by the extreme capsule. Under this model, the superficial layer of this “lamina multiformis” is layer VI of insular cortex, with the deep layer being the claustrum. Similarly, Rose (1928) held that in mammals lacking an extreme capsule, the claustrum is the innermost extension of insular layer VI. In mammals possessing an extreme capsule, both Rose (1928) and Brodmann (1909) suggested that the claustrum is differentiated into an independent cortical layer, with what was termed insular layer VII representing the extreme capsule and layer VIII representing the claustrum. By this definition, the claustrum is cortical, but does not appear layered because it, itself, is a layer of insular cortex. Again, the finding that the claustrum is surrounded by insular cortex cells may have led to the close alignment of the claustrum with the insular cortex by these early researchers.

Standing in opposition to the notion that the claustrum is a cortical component, Landau (1919), and later Faul (1926), believed the claustrum to be subcortical, grouping it with striatal areas, though considering it not to be developmentally related to either striatum or cortex. Holmgren (1925) held a similar view but made the insightful assertion that the claustrum is a pallial structure not derived from cortex. He submitted that the claustrum derives from the ventricular surface, rather than as an in-folding of the overlying insular cortex, and should be grouped along with the amygdaloid complex. His perspective was largely ignored, however, as the bulk of opinions regarded the claustrum as a component part of the insular cortex (Ariëns Kappers et al., 1936, 1960).

It would take 75 years of speculation and investigation before convincing evidence was found to support Holmgren’s view of claustrum ontogeny. Performing an elegant analysis of pallial and subpallial genetic markers in the developing chicken and mouse brains, Puelles et al. (2000) demonstrated the existence of four distinct pallial regions in the developing telencephalon. In addition to the medial, dorsal, and lateral pallial areas previously identified, a new “ventral pallium” was also defined. Based on these findings, Puelles et al. (2000) assigned the claustrum to the lateral pallium, along with the dorsal piriform cortex and basolateral amygdala. The new “ventral pallium” gives rise to the endopiriform nucleus, as well as other sites including the ventral piriform cortex, olfactory bulb, and lateral and intercalated nuclei of the amygdala. This view suggests that because the claustrum lacks a laminar organization and is derived from lateral pallium along with the basolateral amygdala, it should not be considered cortical.

If the claustrum is not a cortical structure, and is derived separately from the endopiriform nucleus, one might predict that the birth date of claustral neurons differs from that of cells in endopiriform nucleus and cortex. Bayer and Altman (1991) used tritiated thymidine birth-dating analysis to determine that rat claustral neurons primarily arise on embryonic day (E) 15 and 16, while endopiriform neurons are born earlier, on E14 and E15. Interestingly, cortical layer VI neurons are born at approximately E12.5, with the more superficial layers completing development by E15.5 (Valverde et al., 1989; Molyneaux et al., 2007). Despite the distinct birth-dating difference between the claustrum and the cortex, Bayer and Altman (1991) showed that claustrum neurons are derived from the cortical epithelium. This finding is consistent with the lateral pallial derivation findings of Puelles et al. (2000), and the position held by Holmgren (1925). In contrast, the endopiriform nucleus derives from the palliostriatal ventricular angle, a zone that straddles the border between the primordia of the basal ganglia and cortex (Bayer and Altman, 1991). Further distinguishing the claustrum from the endopiriform nucleus, claustral neurons migrate ventrally along the axis of the EC where they populate in a caudal to rostral fashion. Endopiriform neurons form a gradient in the orthogonal axis to that of the claustrum, with older neurons populating ventrally, and younger neurons populating dorsally (Bayer and Altman, 1991). So, despite the lack of clear boundaries between the claustrum and the endopiriform nucleus, these structures appear to be developmentally distinct.

## Connectivity

In order to clearly delineate the connections of the claustrum it is imperative to analyze tract tracing data on the basis of an empirical definition of claustrum boundaries. Thus, existing connectivity data will be discussed in light of the Mathur et al. (2009) Gng2/PV-based definition (see Figure 1C).

### Cortical connections

Through the mid-20th century, degeneration studies in rabbit, cat, and macaque (Carman et al., 1964; Narkiewicz, 1964, 1972; Druga, 1966, 1968; Kemp and Powell, 1970; Chadzypanagiotis and Narkiewicz, 1971) suggested that the claustrum is connected with all areas of cortex. A general feature that arose from these studies was that the claustrum is topographically organized, with rostral areas of cortex innervating rostral areas of the claustrum and caudal cortical sites projecting to the more caudal claustrum. Using tract tracing methods, these findings have been substantiated and extended by showing that the cortical projections to the claustrum are reciprocated (Sanides and Buchholtz, 1979; Olson and Graybiel, 1980; Edelstein and Denaro, 2004; Crick and Koch, 2005). Today it is generally accepted that the claustrum is reciprocally connected with all cortical sites (Sherk, 1986), though this position likely requires experimental confirmation. Regardless, it is clear that the claustrum is not equally connected with each cortical area (Alloway et al., 2009; Colechio and Alloway, 2009; Smith and Alloway, 2010). The claustrum appears to project primarily ipsilaterally to the cortex, while a weaker contralateral projection does exist (Norita, 1977; Olson and Graybiel, 1980; Squatrito et al., 1980; Li et al., 1986; Colechio and Alloway, 2009; Mathur et al., 2009; Smith and Alloway, 2010). The reverse appears to be true for cortico-claustral projections, with contralateral projections being denser than their ipsilateral counterparts (Alloway et al., 2009; Smith and Alloway, 2010).

Regarding layer specificity of claustral projections, LeVay (1986) and Olson and Graybiel (1980) showed using a discrete deposit of an anterograde tracer into the claustrum of cat that the claustrum projects to all layers, with the densest innervation to layers IV and VI. Claustral axons synapse with spiny dendrites (of presumptive excitatory cells) in all layers, but in layer IV they also synapse onto aspiny dendrites (LeVay, 1986). Projections from the cortex to claustrum appear to arise predominantly from pyramidal and fusiform cells of layer VI (Olson and Graybiel, 1980; LeVay and Sherk, 1981). Approximately 3–4% of layer VI cells in the visual cortex, for example, project to the claustrum, and this population is distinct from neurons projecting to the lateral geniculate nucleus of the thalamus (Olson and Graybiel, 1980; LeVay and Sherk, 1981). Electron microscopy studies show that cortical projections form asymmetric synapses onto spiny (presumed excitatory) and aspiny (presumed inhibitory) cells of the claustrum (LeVay and Sherk, 1981).

Perhaps the most detailed demonstration of discrete claustral territories is the work done by Olson and Graybiel (1980) in the cat and later confirmed by Remedios et al. (2010) in the rhesus macaque. Olson and Graybiel (1980) used electrophysiological recordings from subregions of the cat claustrum following various sensory stimuli and found that the cortical representation for visual and tactile information within the claustrum maintained an orderly retinotopic and somatotopic organization. By injecting tracers into the claustral site from which they recorded, Olson and Graybiel (1980) found that discrete subdivisions within the claustrum receive projections from and send projections to cognate sensory cortices. In contrast to the rat claustrum, the claustrum of felines and primates, which has expanded along with the cortex, appears far more segregated in its zonal distribution.

Based on the widespread connectivity of claustrum with cortex, and the zones of cortical targeting in the claustrum, it appears that the organization of the claustrum resembles that of the thalamus (Olson and Graybiel, 1980). Are there connections within the claustrum that link these cortical recipient and projection zones together? Following the discrete injections of horseradish peroxidase into the claustrum by Olson and Graybiel (1980) and later LeVay (1986), these investigators reported no inter-zonal connections. However, Smith and Alloway (2010) were able to deposit a retrograde tracer into the rat claustrum and found extensive labeling along the rostro-caudal axis of the claustrum. Further work is needed to completely resolve this issue.

Brodmann (1909), Loo (1931), Rae (1954) and others noted similarities between the insular cortex and the claustrum. The insular cortex, like the claustrum, has widespread connections with other parts of the brain. Studies have shown that the insula projects to or receives inputs from the nucleus of the solitary tract, olfactory bulb, amygdala, hippocampus, the parvicellular part of the posteromedial ventral thalamic nucleus, as well as the entorhinal, motor, primary and secondary somatosensory, prefrontal, orbitofrontal, primary auditory, auditory association, and visual association cortices (Mufson and Mesulam, 1982; van der Kooy et al., 1984; Augustine, 1985, 1996; Nakashima et al., 2000). While the claustrum and the insular cortex share many sites in their respective connectivity profiles, there has been no indication in the literature that these profiles are identical. Based on structural, developmental, and connectivity lines of evidence, the claustrum is not part of insular cortex, despite appearing embedded within layer VI (Mathur et al., 2009).

### Subcortical connections

**Figure 2:**
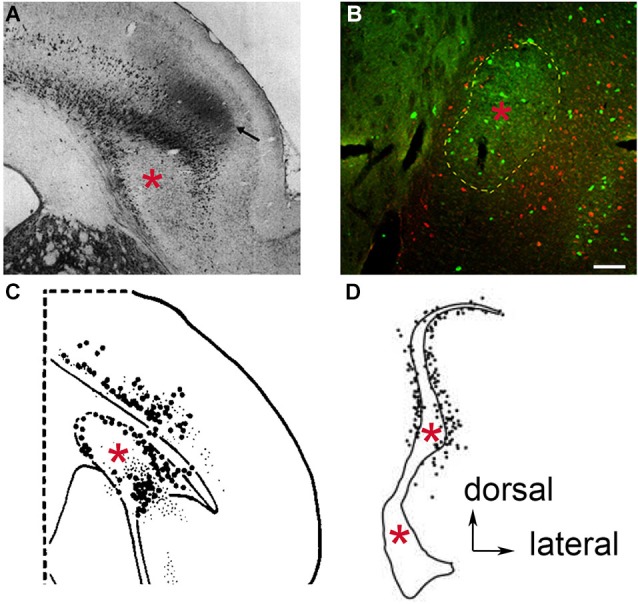
**Retrograde neuronal tract tracer labeling in the region of the claustrum following tracer deposit into the thalamic mediodorsal nucleus of various species**. Examples of species lacking an extreme capsule, **(A)** the hedgehog, (used by permission from John Wiley and Sons; Dinopoulos et al., 1992), and **(B)** the rat, where PV-ir is depicted in green and retrogradely labeled cells in red (see original publication by Mathur et al., 2009). In both cases, the retrogradely labeled cells appear to reside in the insular cortex and surround the body of the claustrum (red asterisk), which in the case of the rat is defined by PV-ir. In species containing an extreme capsule, **(C)** the tree shrew (used by permission from Elsevier; Carey and Neal, 1986), and **(D)** the cynomolgus monkey (used by permission from Elsevier; Erickson et al., 2004) both exhibit a similar pattern of retrograde labeling. The rat data **(B)** suggests that the labeled cells in **(C)** and **(D)** are insular cortex cells that have been separated from the rest of the more superficial insular cortex cell layers through time by the development of the extreme capsule. Scale bar: 100 μm.

In addition to the claustrum’s reciprocal connections with cortex, modern tract tracing studies have suggested the presence of subcortical projections. Studies in the hedgehog, rat, cat, tree shrew, and macaque have reported claustral projections to the dorsal thalamic nuclei (LeVay and Sherk, 1981; Carey and Neal, 1986; Dinopoulos et al., 1992; Erickson et al., 2004; McKenna and Vertes, 2004; Vertes and Hoover, 2008), striatum (Arikuni and Kubota, 1985), hippocampus (Amaral and Cowan, 1980), and hypothalamus (LeVay and Sherk, 1981; Vertes, 1992; Yoshida et al., 2006). Interestingly, in many of these studies the retrogradely-labeled somata in the claustrum were to seen to form a ring-like pattern around the body of the claustrum (Figure 2). These findings can be interpreted as a segregation of the claustrum into a PV-ir rich “core” surrounded by a Vglut2-enriched “shell”, as proposed by Real et al. (2006) in the mouse. According to this, the “shell” may be connected to subcortical sites, while the “core” may be connected with cortex. However, if the “core” and “shell” concept of the claustrum were valid, one might expect that other lines of evidence would distinguish the “shell” from deep layers of insular cortex. However, the rat claustrum was found to be enriched in netrin G-2 protein and cholecystokinin mRNA expression in a pattern consistent with Gng2 expression (Miyashita et al., 2005; Watakabe et al., 2012), while the entire insular cortex was found to be enriched for purkinje cell protein 4 mRNA in a pattern that surrounds the body of the claustrum (Watakabe et al., 2012). Moreover, following retrograde tract tracer injection into subcortical sites, retrogradely-labeled somata surrounding the claustrum typically extend into more superficial layers of the insula as well (Figure 2). The pattern of purkinje cell protein 4 mRNA expression and retrograde labeling throughout the insula further supports that the claustrum is surrounded by insular cortex, rather than being arranged into “core” and “shell” subcomponents that have differential connectivity.

The redefinition of claustral structural boundaries by Gng2/PV expression not only accounts for much of the disagreement over claustrum ontogeny through history, but it also accounts for the abundance of subcortical connection findings. The Gng2/PV-based definition posits the thalamic and lateral hypothalamic connections once believed to be claustral are actually assigned to layer VI of insular cortex. Examination of existing anatomical studies reveals this distinction in several species. Injections of retrograde tracers into the dorsal thalamus of the hedgehog showed retrogradely labeled cell bodies encapsulating the apparent body of claustrum (Dinopoulos et al., 1992; Figure 2A). This is also apparent in rat following injection of retrograde tract tracer into the mediodorsal nucleus of the thalamus (Figure 2B).

Through time, as species developed an extreme capsule following the elaboration of cortex, the claustrum became enveloped by white matter. Prior to the complete encapsulation of the claustrum, this structure was partially bordered laterally by an inchoate extreme capsule in certain species. One example of this is the tree shrew. Injection of a retrograde tract tracer into the dorsal thalamus of this species results in labeled cells again completely surrounding the claustrum notably on the lateral aspect of the claustrum where the extreme capsule borders this structure (Carey and Neal, 1986; Figure 2C). In the macaque, in which the claustrum is completely enveloped in white matter, Erickson et al. (2004) revealed retrogradely-labeled cells surrounding the body of the claustrum again following injection of tracer into the dorsal thalamus (Figure 2D). It should be noted that in these tracer studies, the authors interpreted the findings as a demonstration that the claustrum connects to the dorsal thalamus. However, in light of the Gng2/PV-based definition of claustral boundaries, the retrogradely-labeled cells are likely insular layer VI neurons that populate the perimeter of the white matter-encapsulated claustrum. Interestingly, in the Erickson et al. (2004) study, a large number of retrogradely-labeled cells were observed in the dorsal extension of the macaque claustrum; Crym immunolocalization indicates that this area of the “claustrum” is actually composed of an admixture of insular layer VI and claustral cells (Mathur et al., 2009).

Evidence suggesting the claustrum* receives* subcortical projections is less controversial. Immunohistochemical studies suggest that the claustrum in rats and cats receives a diffuse serotonergic innervation, presumably from the brainstem dorsal raphe nucleus (Baizer, 2001; Rahman and Baizer, 2007). This serotonergic input was reported to be evenly distributed across the entire claustrum (Rahman and Baizer, 2007). Consistent with these findings, some evidence exists for expression of five subtypes of serotonin receptors within the claustrum, including 5-HT_1A_, 5-HT_1F_, 5-HT_2A_, and 5-HT_2C_ receptors (Pompeiano et al., 1994; Wright et al., 1995; Mengod et al., 1996; Pasqualetti et al., 1999). The significance of this potential subcortical connection has yet to be experimentally elucidated.

Another subcortical structure that has been reported to project to the claustrum is the endopiriform nucleus, which lies immediately ventral to the claustrum. Lipowska et al. (2000) found that the endopiriform nucleus in the rat and rabbit projects to the perimeter of the claustrum. This connectivity pattern would again appear to be consistent with the notion of a “core” and “shell” organization of the claustrum. Thus, the “shell” of the claustrum projects to endopiriform nucleus. Alternatively, and in accordance with the Gng2/PV-based definition of the claustrum, these projections to the endopiriform nucleus arise instead from the insular cortex. It should be noted that discrete tracer injections into the endopiriform nucleus are extremely difficult to achieve. Because the endopiriform nucleus borders white matter, claustrum, and insula, endopiriform connectivity data based on tract tracing carries a note of caution.

## Function

The final, and most puzzling, “problem” of the claustrum lies in its function. Relative to other prominent telencephalic structures such as the cortex, striatum, and thalamus, knowledge of claustral function is sorely lacking. Despite waves of interest in the claustrum over the last century, only a few nuggets of functional information and some controversial hypotheses on its functional attributes exist. Why has the function of the claustrum proven to be so hard to unlock? The shape of the claustrum has made complete and discrete claustrum lesions impossible to achieve using conventional chemical or mechanical means. Clinical pathological correlation studies have yielded extraordinary information about the function of many brain sites, but no convincing selective claustral lesions have been reported in humans following cerebral hemorrhage or ischemia. Without the ability to generate reproducible, discrete lesions of the claustrum in animals, the functional roles of this nucleus remain a mystery.

### Multisensory integration

Based on its bidirectional cortical connectivity, the claustrum has been proposed to function as a multisensory integrator; serving to bind information from disparate sensory cortices. Supporting this notion, Segundo and Machne (1956) and later Spector et al. (1974) found electrophysiological evidence for sensory convergence in the claustrum. Both groups recorded from claustral neurons in awake and anesthetized cats that were exposed to sensory stimuli of different modalities. They showed that 75% claustral cells responded to more than one sensory modality (Spector et al., 1974). The polymodal neurons responded to as few as two modalities, and to as many as six (touches, flashes, clicks, smells, vagal, and tooth pulp stimulation). The most common convergences observed were somato-olfactory, somato-visceral, and somato-nocioceptive (Segundo and Machne, 1956). Polymodal cells were distributed throughout the claustrum (Spector et al., 1974), and these cells displayed unique firing patterns for each type of modality-specific stimulus (Segundo and Machne, 1956). Given that the claustrum may be surrounded/intermingled with insular cortical cells (Mathur et al., 2009), these previous studies are called into question.

Two different theories for multisensory integration have been proposed. The first theory states that multisensory integration occurs in polymodal sites that only process specific sensory combinations; these types of cells have been reported in a variety of areas including arcuate sulcus, superior temporal sulcus, inferior and posterior parietal lobules, the amygdaloid complex, hippocampus, and the superior colliculus (Thompson and Shaw, 1965; Ettlinger and Wilson, 1990). Because the claustrum appears to have multisensory-responsive cells, the claustrum may serve to bind some types of sensory modalities. The second theory, proposed by Ettlinger and Wilson (1990), states that no one structure in brain executes the processes required for cross-modal performance. Instead, only a subcortical relay nucleus is required through which different sensory cortices can access each other in order to associate modalities. This subcortical relay nucleus was proposed to be the claustrum. In this way, the claustrum theoretically synchronizes cortical areas to accomplish the feat of crossing modalities. Ettlinger and Wilson (1990) did not state, however, how this may be accomplished or where the binding of multimodal information would occur.

*In vivo* functional imaging studies exploring multisensory integration largely support the second theory, which places the claustrum as the necessary subcortical relay nucleus. This support is due to a growing body of evidence showing activation of the claustrum/insula region in cross-modal matching tasks (Hörster et al., 1989; Lewis et al., 2000; Olson et al., 2002; Naghavi et al., 2007; Kavounoudias et al., 2008). A possible representative finding comes from Hadjikhani and Roland (1998) positron emission tomography (PET) study that involved a task that had subjects attempting to identify objects in their hand (to which they were blind) to a matching object in their visual field (but out of reach) that was amongst a series of similar, but non-identically-shaped objects. They found that the insula-claustrum region, with a center of gravity situated closer to the claustrum, was the only area constantly activated in these tasks. A caveat with this study is that the claustrum and insula are impossible to distinguish with the imaging resolution provided by PET. Other studies using functional magnetic resonance imaging (fMRI) have gone on to show that a combination of the appropriate sensory cortices and the claustrum were activated during similar matching paradigms (Olson et al., 2002; Naghavi et al., 2007; Kavounoudias et al., 2008). Thus, a relay function for the claustrum enjoys support.

Arguing against multisensory integration is recent work by Remedios et al. (2010) who improved targeting of the claustrum using magnetic resonance to guide the placement of the recording electrode in awake monkeys. They found that claustral neurons are relatively quiescent and, supporting the earlier work by Olson and Graybiel (1980), that the claustrum is subdivided into discrete sensory zones. That is, the visual zone of the claustrum preferentially and transiently responds to suddenly presented visual cues, while the auditory subdivision of the claustrum does the same for auditory cues (Remedios et al., 2010). Polymodal responses were rarely observed.

The imaging studies that do support a role for the claustrum in multisensory integration do not address the question of where polymodal information is being bound exactly, and again suffer from the inability to discriminate between claustral vs. insular activation. Moreover, it is quite possible that, given the negative multisensory findings by Remedios et al. (2010) that claustral activation under multisensory tasks may be an effect of activity pooling, wherein the BOLD activity threshold for the claustrum is achieved only when multiple, discrete sensory zones of the claustrum are simultaneously activated.

### Crick and Koch’s hypothesis

Crick and Koch (2005) hypothesized that the claustrum is where sensory information is bound, functioning as a generator of the unified perception of a multitude of sensory stimuli in one’s environment (conscious percepts). That is, putting individual stimuli together, one is able to recognize an object as a whole rather than experiencing each stimulus as a separate sensory entity. Crick and Koch argued that since almost all theories attempting to explain the neural correlate of such an experience (consciousness) require a “need to rapidly integrate and bind information in neurons that are situated across distinct cortical and thalamic regions” (see also Bachmann, 2000; Llinas, 2001), that the claustrum may be perfectly suited to subserve such a function due to its unique feature of reciprocal connectivity with the cortex, its central positioning in brain, and its connections with the thalamus (which are now called into question, see Mathur et al., 2009). Crick and Koch (2005) went on to propose that the binding of multisensory information in the claustrum underlies the unification of sensory experiences. This hypothesis has received further theoretical support from Smythies et al. (2012), who propose that the claustrum functions as a detector, modulator, and integrator of synchronous oscillations for the purpose of subserving cognitive processes such as consciousness.

Though the claustrum does appear to have many of the attributes required of a sensory binding site, some problems exist with this concept. First, a well-recognized physiological trait of claustral cells consistently found across functional studies is their quiescent nature (Segundo and Machne, 1956; Spector et al., 1974). The spontaneous firing rate is quite low, usually only becoming activated following the presentation of a sudden sensory stimulus in awake monkeys (Remedios et al., 2010). If the claustrum is binding sensory stimuli for the purpose of generating conscious percepts, one would predict that the claustrum would display near constant activation during awake, behaving conditions. Secondly, the Crick and Koch model places the high computational load requirement of binding in a structure that is not layered, or at least not organized (by sensory subdivisions) in such a way that would suggest processing power as we currently know it.

### From structure to function?

The Gng2/PV-based anatomical definition of the claustrum indicates this structure may be largely restricted to (reciprocal) connections with cortical sites, and does not project subcortically to structures including the lateral hypothalamus and mediodorsal thalamic nucleus (Mathur et al., 2009). Given the normally quiescent nature of claustral cells that respond transiently to suddenly presented stimuli then (Remedios et al., 2010), the claustrum could serve as a saliency filter for cortico-cortico communication. Given that the claustrum preferentially projects to the ipsilateral anterior cingulate cortex (Smith and Alloway, 2010), an area known to be involved in error detection and attentional processing (Muir et al., 1996; Botvinick, 2007; Carter and van Veen, 2007; Johnston et al., 2007), the claustrum may be acting as a component part of a sensorimotor/sensory association cortex-to-claustrum-to-cingulate pathway for encoding the salience of incoming stimuli. This would allow only the most salient signals to propagate through the claustrum to the cingulate cortex. It is also possible that a cingulate cortex-to-claustrum-to-sensorimotor cortex/association cortex circuit may be recruited for allocation of attentional load to the necessary cortical sites demanded of a particularly salient stimulus. These hypotheses could possibly be tested using optogenetic activation/inactivation of cingulate fibers projecting to the claustrum during a modified 5-choice reaction test in rodents. This is the standard assay for assessing attentional ability in rodents. It involves presenting a brief light stimulus in one of five possible holes arrayed in a horizontal arc in front of the animal. Once the cue is presented, the animal must successfully nose-poke the hole where the light flashed and a food reward is delivered. The task basically tests the ability to sustain attention to a number of locations over a series of trials (Robbins, 2002).

The saliency detection concept fits with existing structural and functional data and presents testable predictions. The first prediction is that the claustrum is most intimately connected to higher order association cortices, rather than primary sensory cortices. If the claustrum is indeed involved in attention, then attentional resources should be allocated to cortices encoding for complex representations (faces) rather than those encoding the component parts of objects (lines). This could easily be tested using a series of traditional anterograde and retrograde neuronal tract tracer injections into the necessary cortical sites. The second prediction would be that the claustrum would be expected to be recruited during tasks requiring attentional shifting or the early phase of attentional focusing. Based on connectivity studies over several decades, including recent work in rats showing that claustro-cortical connections are significantly stronger ipsilaterally and cortico-claustral projections display the opposite configuration (Alloway et al., 2009; Colechio and Alloway, 2009; Mathur et al., 2009; Smith and Alloway, 2010), one could then hypothesize that claustral activation may be commonly observed to be unilateral, unless the salient stimulus was presented bilaterally. This could possibly be tested using functional imaging in humans (given sufficient resolution of the claustrum) or *in vivo* electrophysiology in non-human primate subjects presented with unilateral vs. bilateral stimuli during attentional tasks.

**Figure 3:**
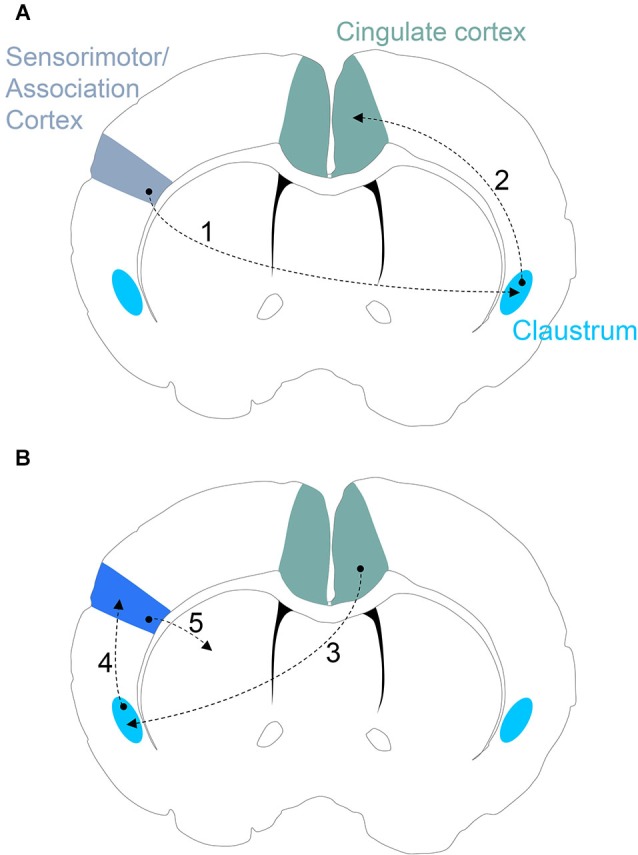
**(A)** Proposed claustrum circuitry involved in stimulus encoding. Neural activity encoding a novel/salient sensory stimulus in sensorimotor and/or association cortices activates the corresponding subdivision of the contralateral claustrum (*Step 1*). If the sensory stimulus is salient enough to pass the claustral filter, the ipsilateral cingulate cortex receives and processes the incoming claustral signal (*Step 2*). **(B)** Proposed circuitry involved in an action response to a salient stimulus. The cingulate cortex signals to the appropriate subdivision of the contralateral claustrum (*Step 3*) that, in turn, provides attentional allocation to the original sensorimotor/association cortex encoding the salient stimulus (*Step 4*). The activated sensorimotor/association cortex finally signals to the striatal complex for selection of an appropriate action (*Step 5*).

For an incoming, salient sensory stimulus encoded by sensorimotor or association cortices, once a certain threshold of salience is achieved, the contralateral claustrum would be activated (Figure 3). Claustral activation would signal to ipsilateral cingulate cortex. Through the claustrum, then, the cingulate cortex enjoys an online saliency map of the cortical mantle. Cingulate processing may then result in contralateral activation of claustrum that would, in turn, result in claustral activation of the original sensorimotor or association cortical site for the allocation of attentional demand to the perceived salient stimulus (Figure 3). The zonal organization of cortical representation in the claustrum again becomes necessary in this context. Using this organization, the cingulate cortex channels signals through the appropriate claustrum sensory subdivision to prime the cognate sensorimotor/association cortical site. If the claustrum was not arranged into discrete zones, and these sensory subdivisions were intermixed, it would seem likely that the categorical allocation of attention to distinct sensory cortices would be blurred.

If the claustrum is functioning as a component part of a distributed network for attentional allocation, a role for this circuit in instigating cortico-basal ganglia circuitry for action selection can be envisioned. In response to a salient stimulus, claustral activation of a sensorimotor/association cortex may prime subsets of corticostriatal circuits for initiation/selection of a particular action (or inaction) through enhanced synaptic drive or synchrony at select corticostriatal synapses. It is also plausible that claustral priming of a select corticostriatal circuit may enhance learning of a motor sequence or skill response to a salient stimulus. As novel stimuli are often perceived as salient, the claustrum may also be involved in attentional allocation to cortical sites signaling to the striatal complex during the learning of novel actions. These predictions could be tested using optogenetic manipulation of claustral afferents to cortical sites projecting to the striatum, such as motor cortex, during acquisition of a skill.

## Conclusions

The expression of Gng2 and PV immunoreactivity offers an empirical definition of claustral boundaries and describes the relationship of the claustrum to contiguous structures. In doing so, the Gng2/PV-based definition challenges the currently held view of claustral connectivity. That is, the claustrum appears to connect (reciprocally) to cortex, and not to project to other prominent subcortical sites (lateral hypothalamus and mediodorsal nucleus of the thalamus) as once thought (Mathur et al., 2009). However, afferent connections from subcortical sites such as the dorsal raphe nucleus remain a possibility.

It is clear that a consensus on the structural boundaries of the claustrum is required. Such a consensus would result in agreement on the claustrum’s connectivity profile and, in turn, shed light on the possible functional attributes of this nucleus. Towards this end, it is imperative that mouse lines expressing green fluorescent protein or Cre recombinase under control of a claustral-specific gene (e.g., Gng2) promoter are generated to allow for the next generation of cell type classification, functional characterization and microcircuit mapping of the claustrum. Such tools would allow for *in vivo* control of claustral activation with light, ultimately providing a long-sought and elegant means of testing existing and future functional hypotheses of the “problem” that is the claustrum.

## Conflict of interest statement

The author declares that the research was conducted in the absence of any commercial or financial relationships that could be construed as a potential conflict of interest.
